# Supplementary material for: Synthesis and Biological Properties of Fluorescent Strigolactone Mimics Derived from 1,8-Naphthalimide
Source: Molecules. 2024 May 12;29(10):2283. doi: 10.3390/molecules29102283 (PMC11124091; doi:10.3390/molecules29102283)
Supplement: Supplementary file 1 [file molecules-29-02283-s001.zip › molecules-2959537-supplementary.pdf]

Supplementary Material

Synthesis and Biological Properties of Fluorescent Strigolactone Mimics derived from 1,8-Naphthalimide

by Ioana-Alexandra Bala, Alina Nicolescu, Florentina Georgescu, Florea Dumitrascu, Anton Airinei, Radu Tigoianu, Emilian Georgescu, Diana Constantinescu-Aruxandei, Florin Oancea, Calin Deleanu

Content

1. NMR spectroscopy 1
2. MS spectrometry 6
3. Photophysical properties 7
4. Microscopy 9

1. NMR spectroscopy

Please note that the ^{15}N correlation H,N-HMBC and the H,H COSY spectra for compound **7** (**SL26**) have been included in the main part of the paper (**figures 4a and 4b**) and they are not repeated here.

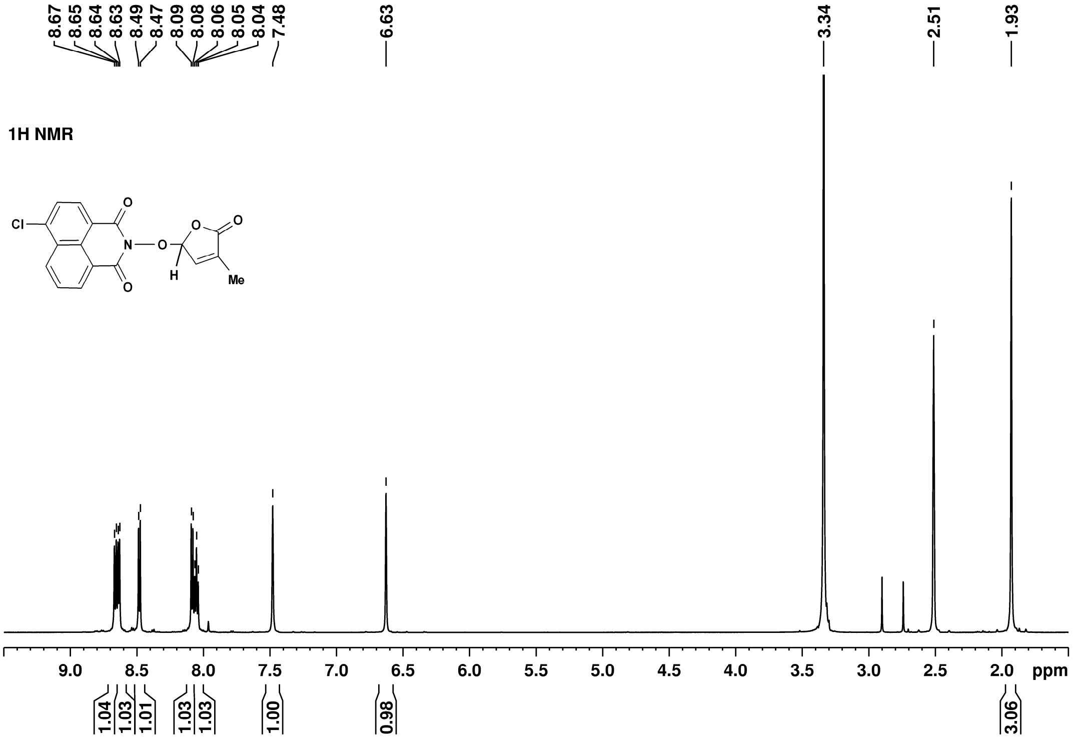

Figure S1. ^{1}H NMR spectrum corresponding to SL mimic **7** (**SL26**), recorded in DMSO-d6, at 600 MHz.

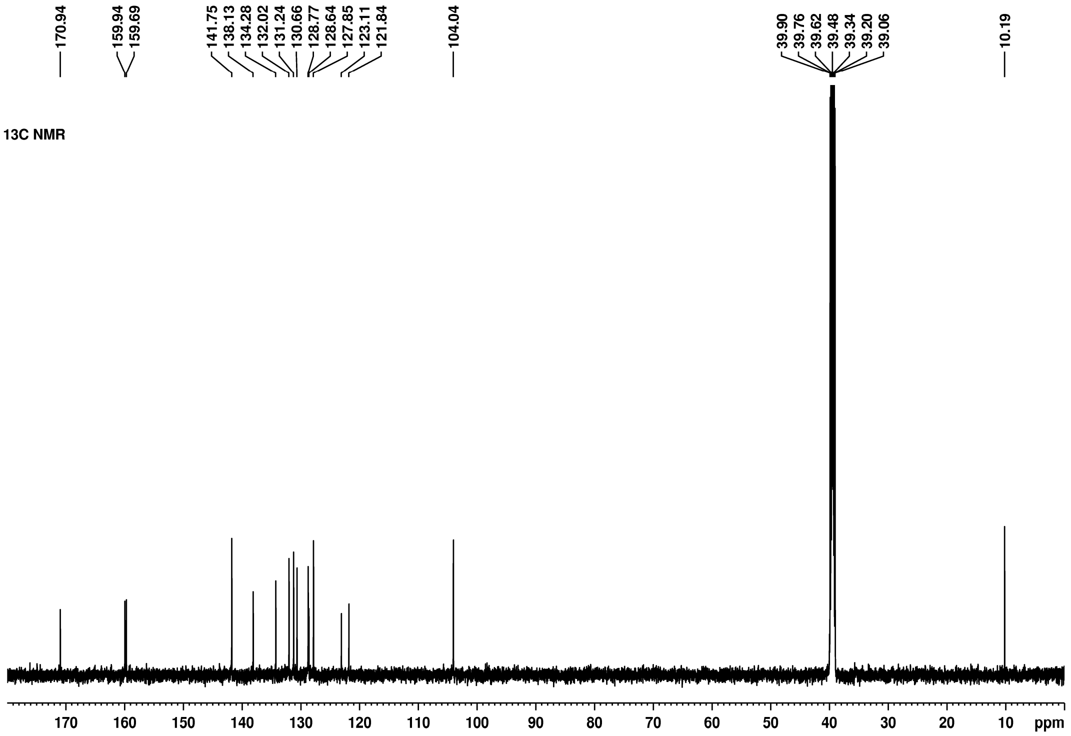

Figure S2. ^{13}C NMR spectrum corresponding to SL mimic **7** (**SL26**), recorded in DMSO-d6, at 150 MHz.

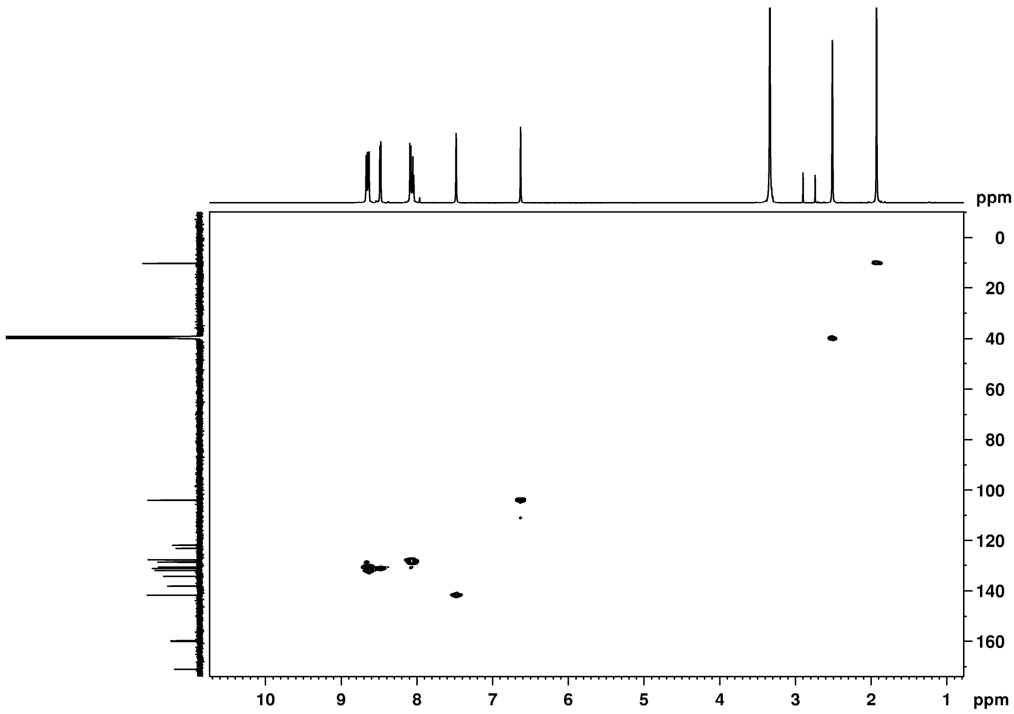

Figure S3. H,C HSQC spectrum corresponding to SL mimic **7** (**SL26**), recorded in DMSO-d6, at 600 MHz.

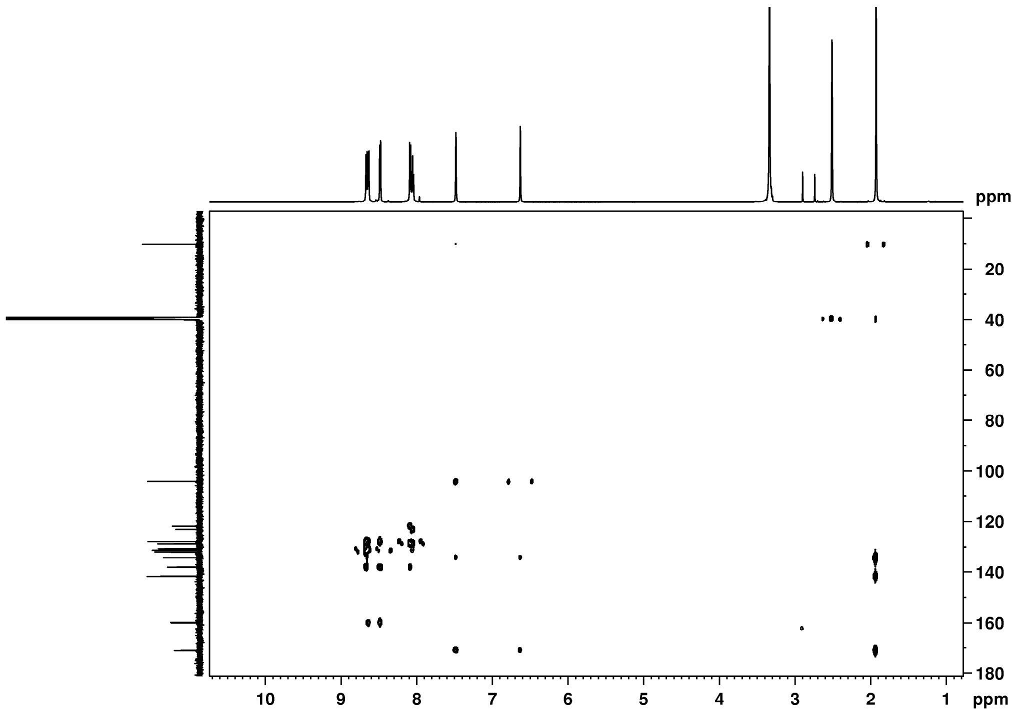

Figure S4. H,C HMBC spectrum corresponding to SL mimic **7** (**SL26**), recorded in DMSO-d6, at 600 MHz.

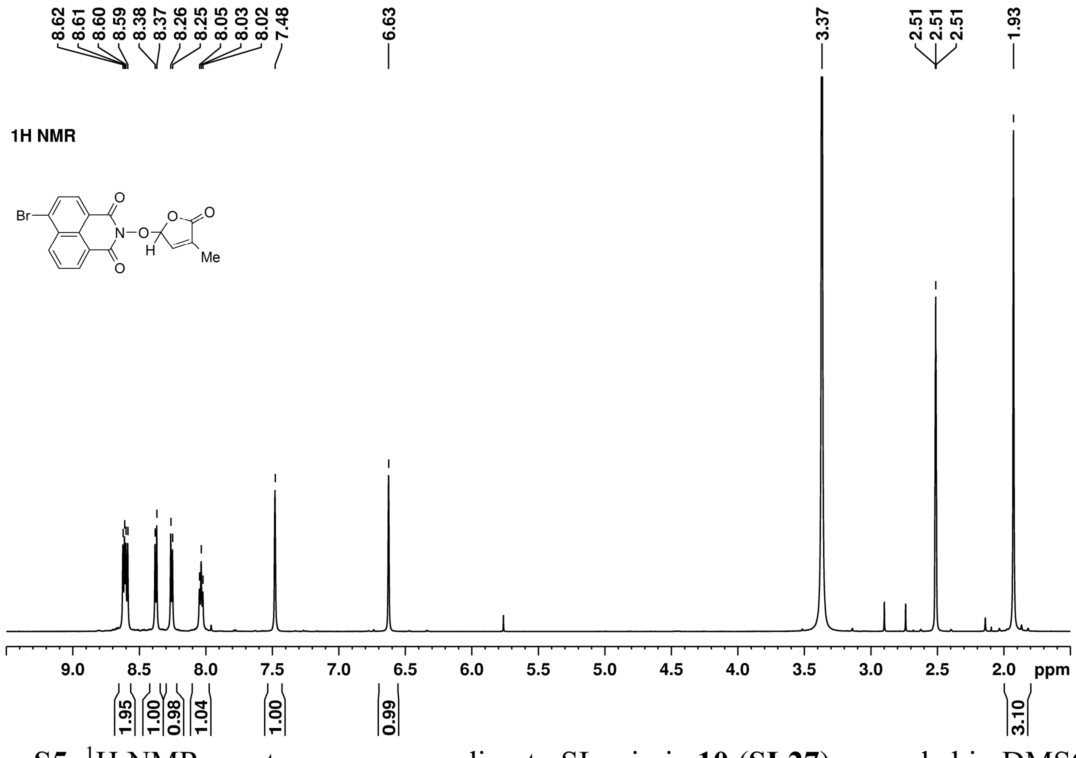

Figure S5. ^{1}H NMR spectrum corresponding to SL mimic **10** (**SL27**), recorded in DMSO-d6, at 600 MHz.

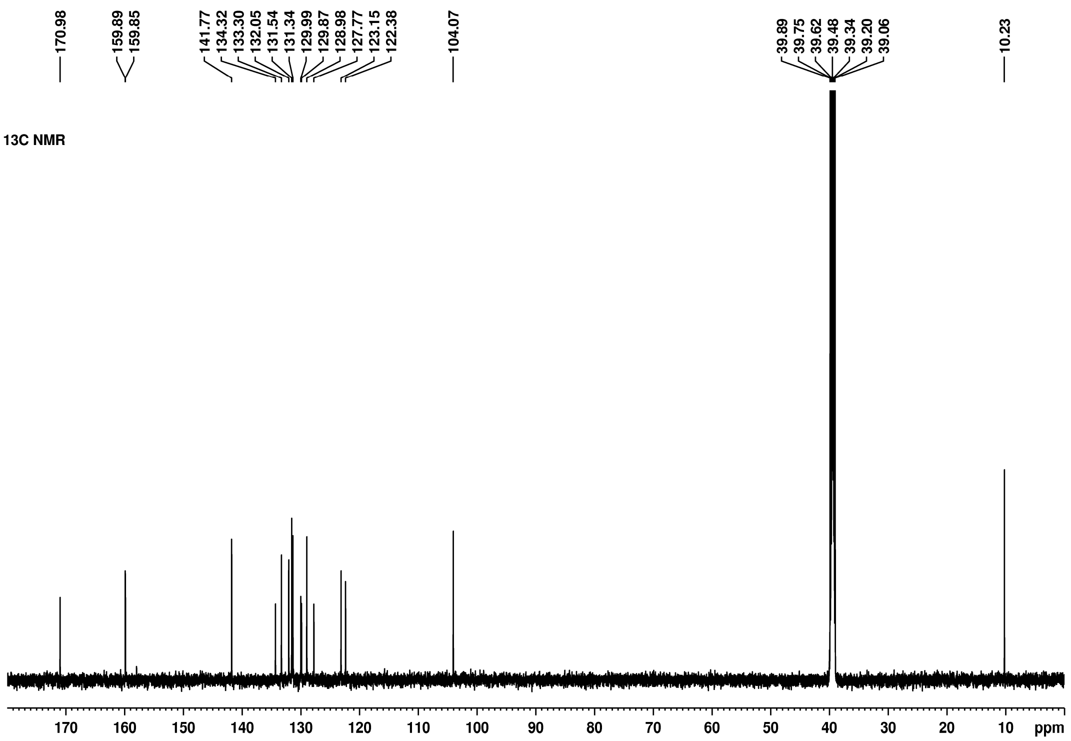

Figure S6. ^{13}C NMR spectrum corresponding to SL mimic **10** (**SL27**), recorded in DMSO-d6, at 150 MHz.

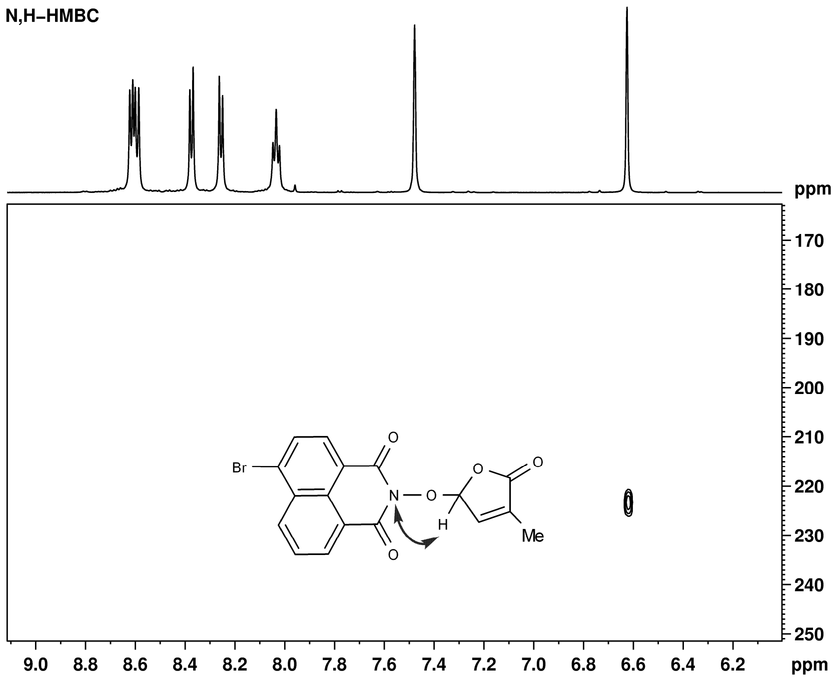

Figure S7. H,N HMBC spectrum corresponding to SL mimic **10** (**SL27**), recorded in DMSO-d6, at 60.8 MHz.

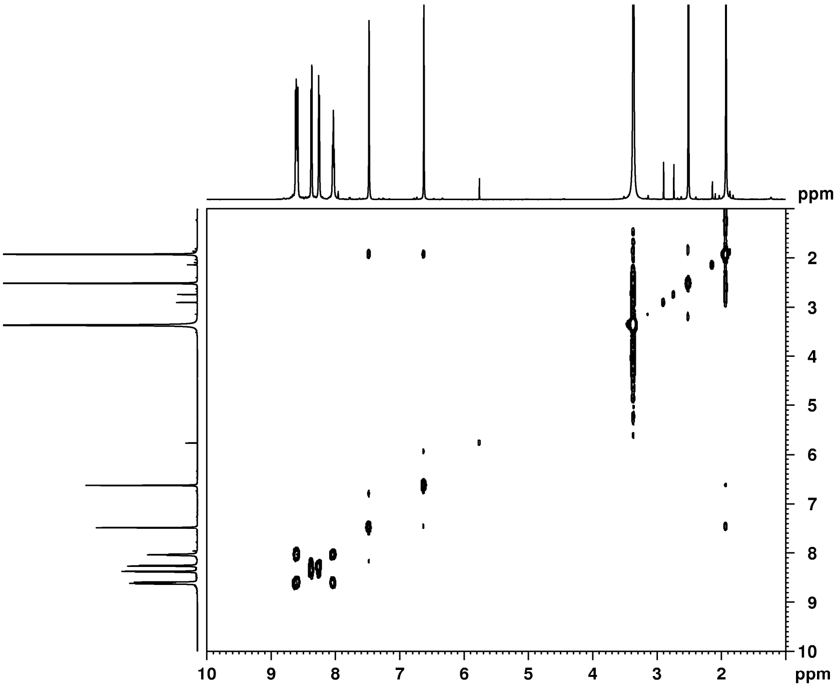

Figure S8. H,H COSY spectrum corresponding to SL mimic **10** (**SL27**), recorded in DMSO-d6, at 600 MHz.

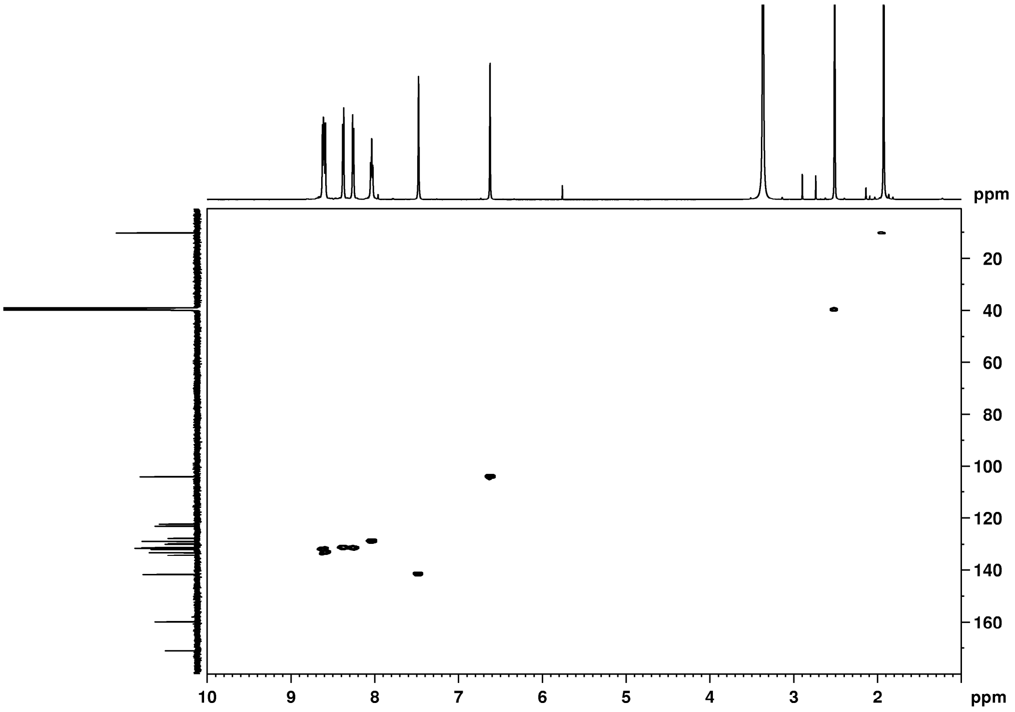

Figure S9. H,C HSQC spectrum corresponding to SL mimic **10** (**SL27**), recorded in DMSO-d6, at 600 MHz.

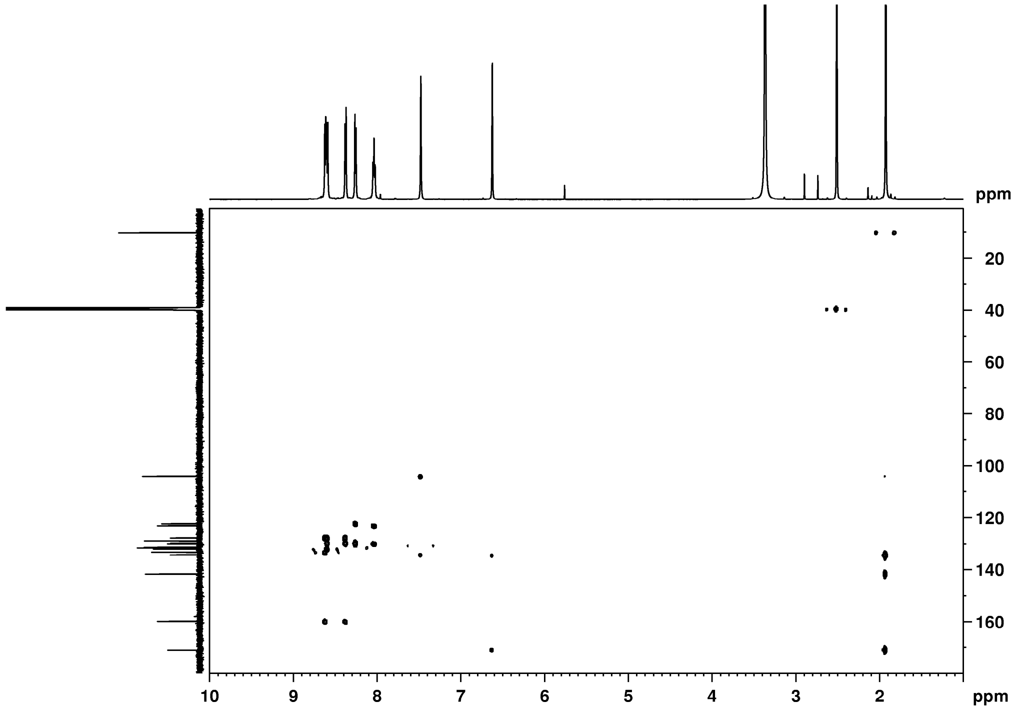

Figure S10. H,C HMBC spectrum corresponding to SL mimic **10** (**SL27**), recorded in DMSO-d6, at 600 MHz.

2. MS spectrometry

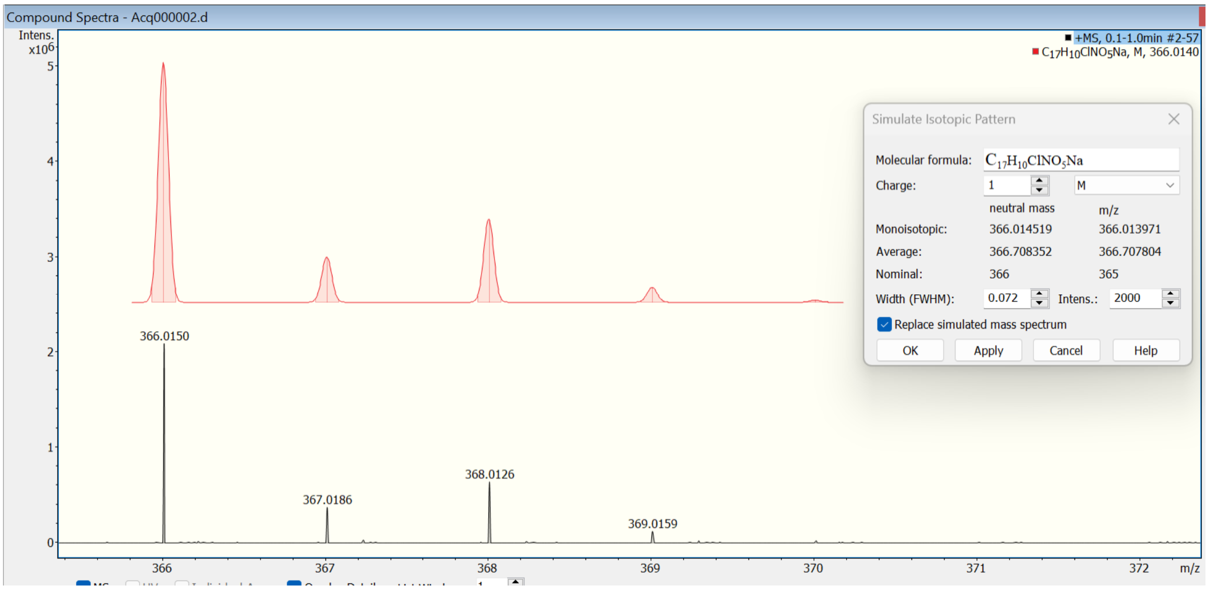

Figure S11. HRMS-ESI spectrum with isotopic pattern in positive mode for SL mimic **7** (**SL26**). Upper trace (red) – simulated pattern, lower trace (black) – experimental spectrum for $C_{17}H_{10}BrNO_5$ $[M+Na]^+$. HRMS-ESI (m/z): $[M+Na]^+$ for $C_{17}H_{10}ClNO_5$, calcd. 366.0145, found 366.0150.

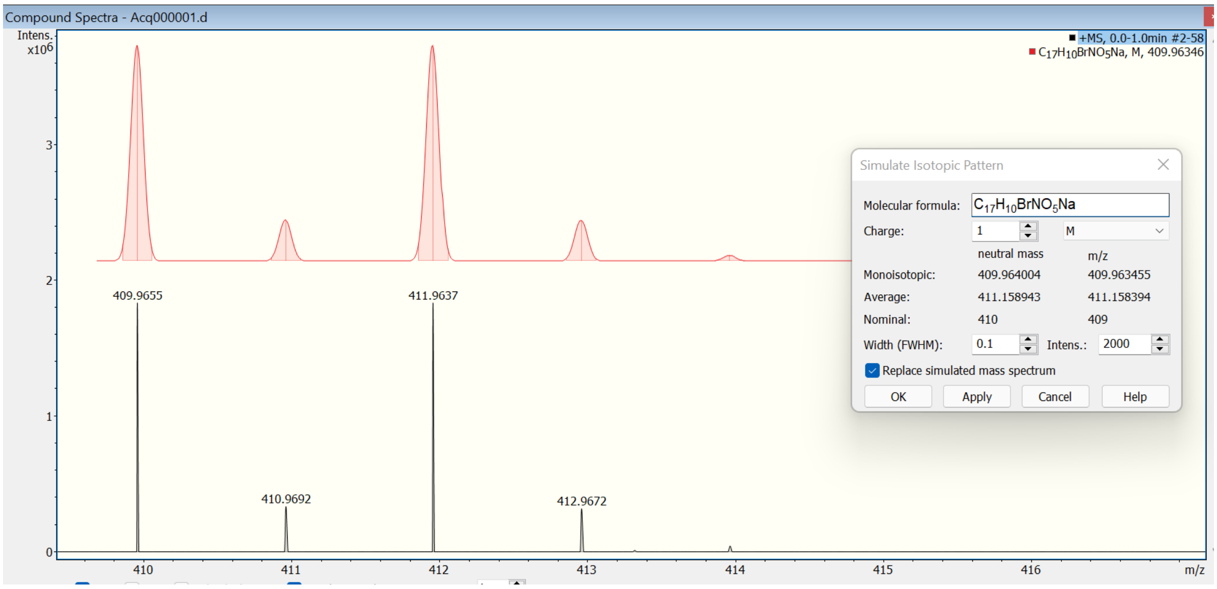

Figure S12. HRMS-ESI spectrum with isotopic pattern in positive mode for SL mimic **10** (**SL27**). Upper trace (red) – simulated pattern, lower trace (black) – experimental spectrum for $C_{17}H_{10}BrNO_5$ $[M+Na]^+$. HRMS-ESI (m/z): $[M+Na]^+$ for $C_{17}H_{10}BrNO_5$, calcd. 409.9640, found 409.9655.

3. Photophysical properties

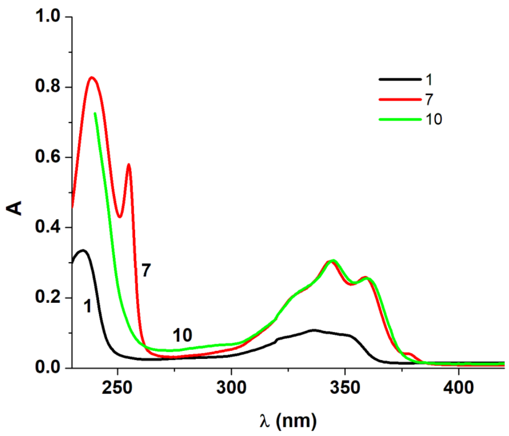

Figure S13. Absorption spectra of the SL mimics **1** (**SL6**), **7** (**SL26**), and **10** (**SL27**) in dichloromethane.

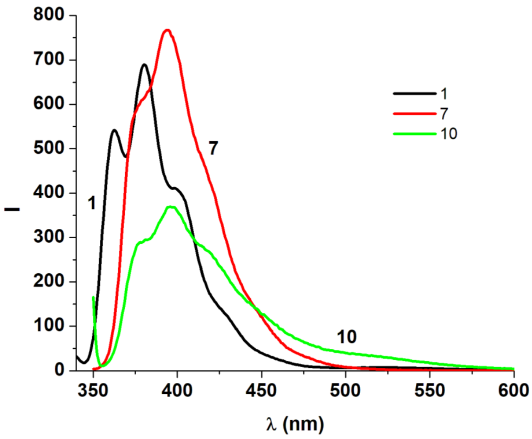

Figure S14. Emission spectra of the SL mimics **1** (**SL6**), **7** (**SL26**) and **10** (**SL27**) in dichloromethane.

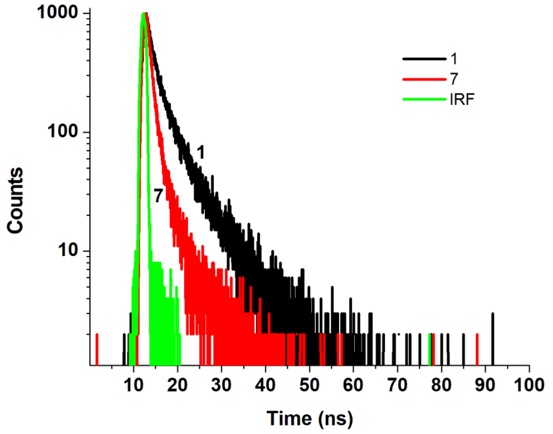

Figure S15. The fluorescence decay traces of SL mimics **1 (SL6)** and **7 (SL26)** in toluene. IRF-Instrument Response Function

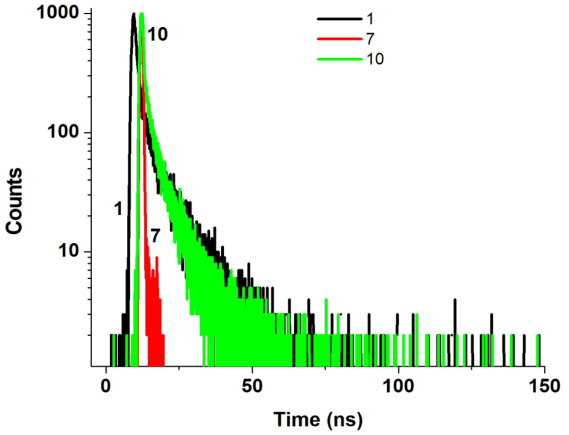

Figure S16. The fluorescence decay traces of SL mimics **1** (**SL6**), **7** (**SL26**) and **10** (**SL27**) in DMF.

Table S1. Spectral and photophysical characteristics of the SL mimics **1**, **2** and **3** measured in different organic solvents.

Sample	Solvent	λ_{max} (nm)	λ_{em} (nm)	$\Delta\upsilon$ (cm^{-1})	Φ (%)
**1** [69]	PhMe	320sh[1], 335, 350	403.8	5085	1.86
	DCM	320sh, 336, 350sh	362.3, 381, 399.5	3506	25.67
	DMF	325sh, 336, 350sh	377.7	3284	0.01
**2** [70]	PhMe	329, 344, 407	497.6	4409	54.63
	DCM	261, 281, 325sh, 343, 422	521.5	4519	45.14
	DMF	325sh, 342, 418	528.0	4881	21.42
**3** [70]	PhMe	328, 344, 406	496.4	4514	45.27
	DCM	260, 280, 325sh, 343, 420	520.3	4589	52.99
	DMF	325sh, 342, 418	535.9	5263	6.37

[1]sh – shoulder; PhMe: toluene; DCM: dichloromethane; DMF: dimethylformamide; λ_{max}: absoption maximum wavelength; λ_{em}: emission wavelength; Δv: Stokes shifts; Φ: quantum yield

Table S2. Fluorescence lifetimes of the SL mimics **1**, **2** and **3**.

Sample	Solvent	τ_1 (ns)	τ_2 (ns)	τ_3 (ns)	a_1 (%)	a_2 (%)	a_3 (%)
**1** [69]	PhMe	1.49	5.99	-	48.44	51.56	-
	DMF	0.30	1.26	5.09	35.75	35.34	28.91
**2** [70]	PhMe	7.96	-	-	100	-	-
	DCM	8.36	-	-	100	-	-
	DMF	0.23	6.42	-	39.72	60.28	-
**3** [70]	PhMe	7.75	-	-	100	-	-
	DCM	8.43	-	-	100	-	-
	DMF	0.18	5.95	-	78.81	21.19	-

4. Microscopy

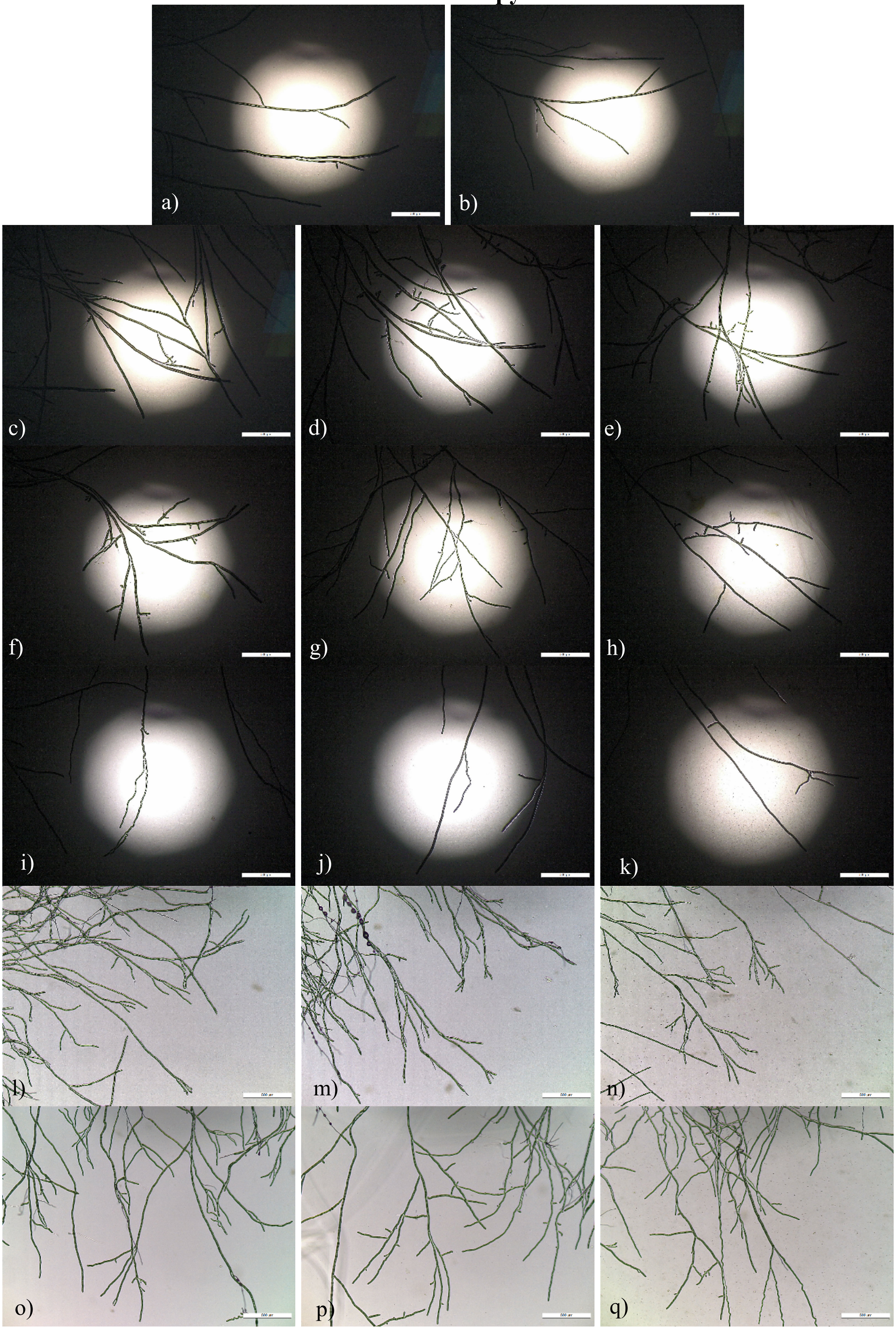

Figure S17. The aspect of hyphal branching of *Fusarium graminearum* in a) control water agar, b) acetone, c) GR24 C1, d) GR24 C2, e) GR24 C3, f) SL20 C1, g) SL20 C2, h) SL20 C3, i) SL21 C1, j) SL21 C2, k) SL21 C3, l) SL26 C1, m) SL26 C2, n) SL26 C3, o) SL27 C1, p) SL27 C2, q) SL27 C3 recorded on 2000 μm beginning from the end of the youngest hyphal edge.

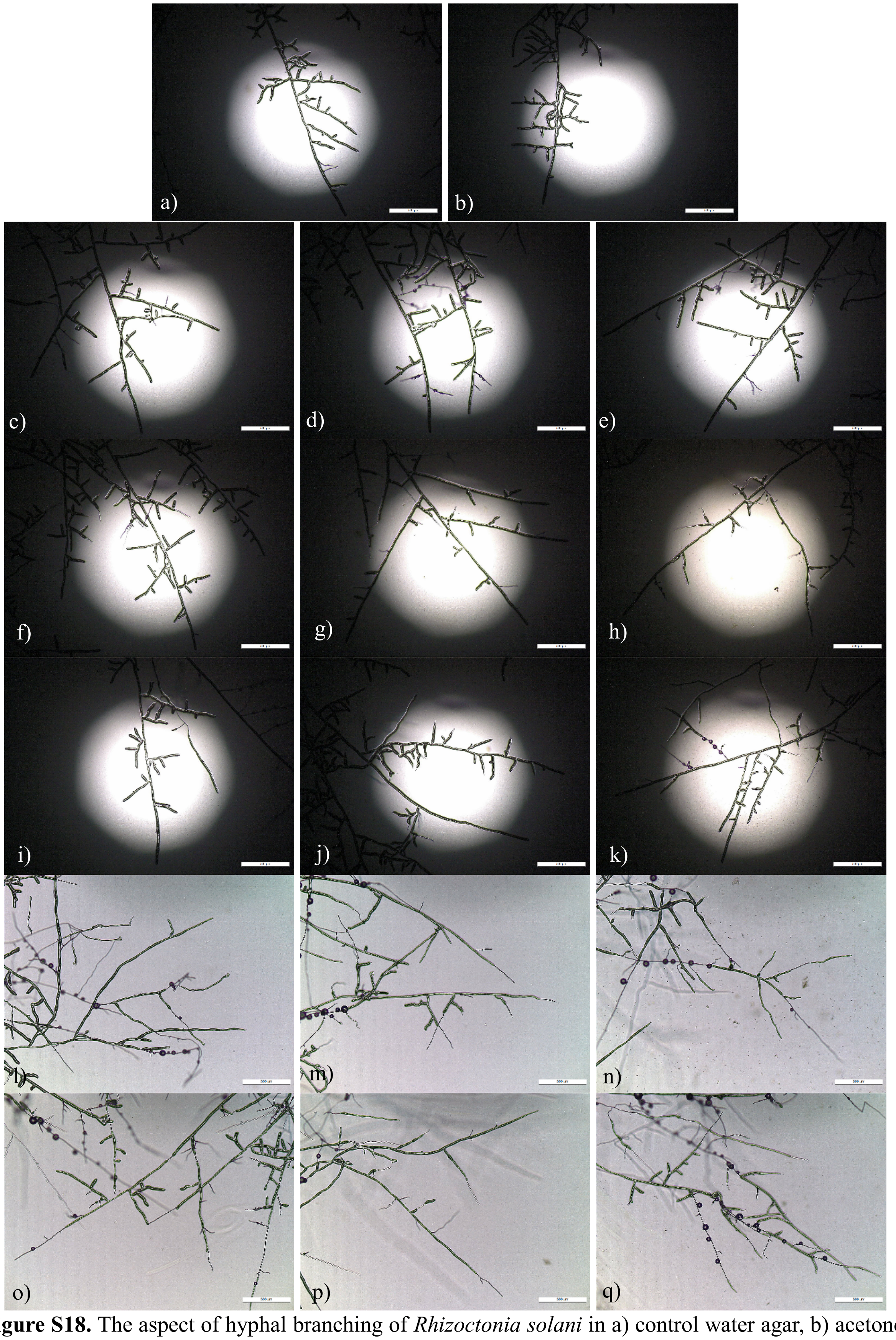

Figure S18. The aspect of hyphal branching of *Rhizoctonia solani* in a) control water agar, b) acetone, c) GR24 C1, d) GR24 C2, e) GR24 C3, f) SL20 C1, g) SL20 C2, h) SL20 C3, i) SL21 C1, j) SL21 C2, k) SL21 C3, l) SL26 C1, m) SL26 C2, n) SL26 C3, o) SL27 C1, p) SL27 C2, q) SL27 C3 recorded on 2000 μm beginning from the end of the youngest hyphal edge.

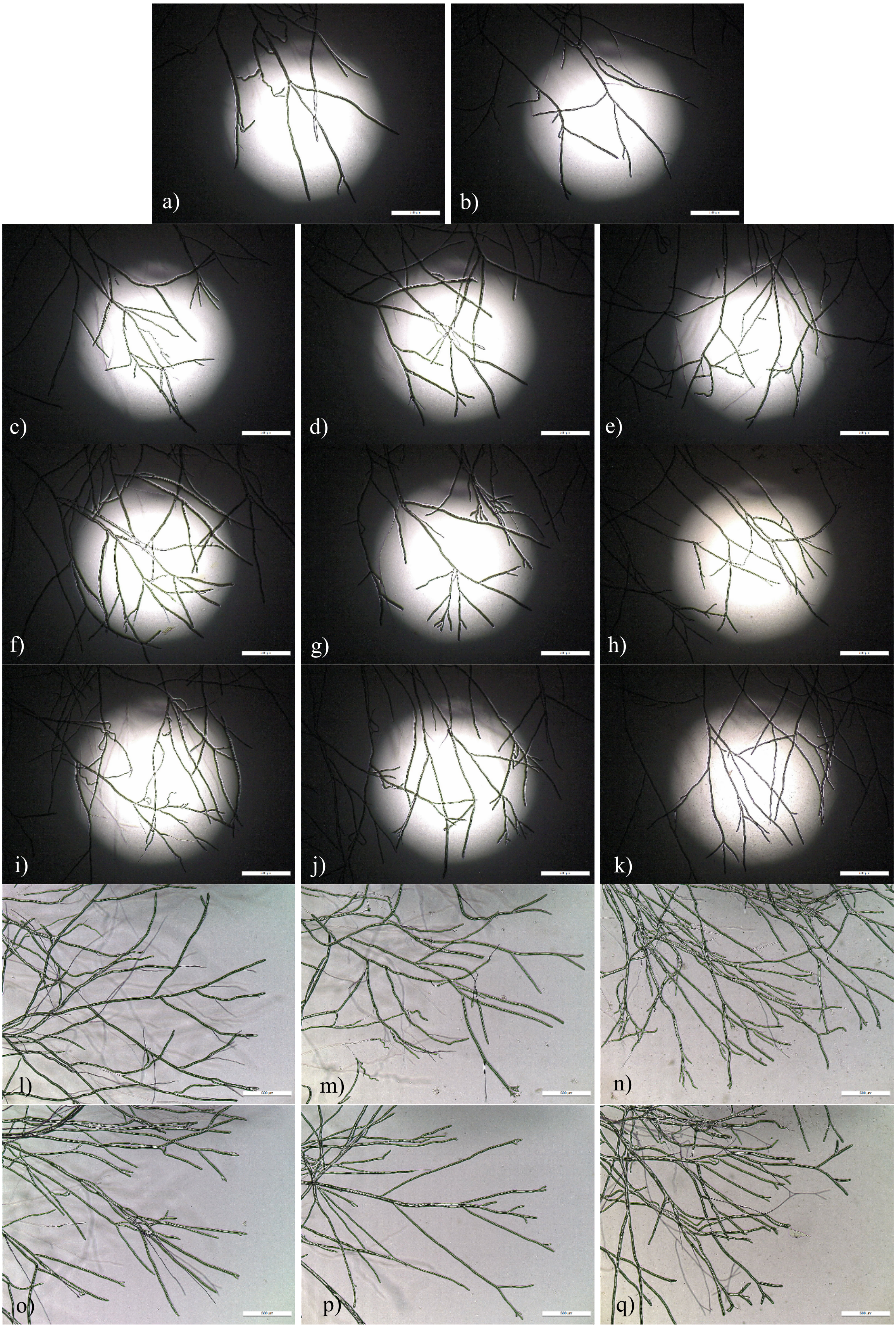

Figure S19. The aspect of hyphal branching of *Sclerotinia sclerotium* in a) control water agar, b) acetone, c) GR24 C1, d) GR24 C2, e) GR24 C3, f) SL20 C1, g) SL20 C2, h) SL20 C3, i) SL21 C1, j) SL21 C2, k) SL21 C3, l) SL26 C1, m) SL26 C2, n) SL26 C3, o) SL27 C1, p) SL27 C2, q) SL27 C3 recorded on 2000 μm beginning from the end of the youngest hyphal edge.

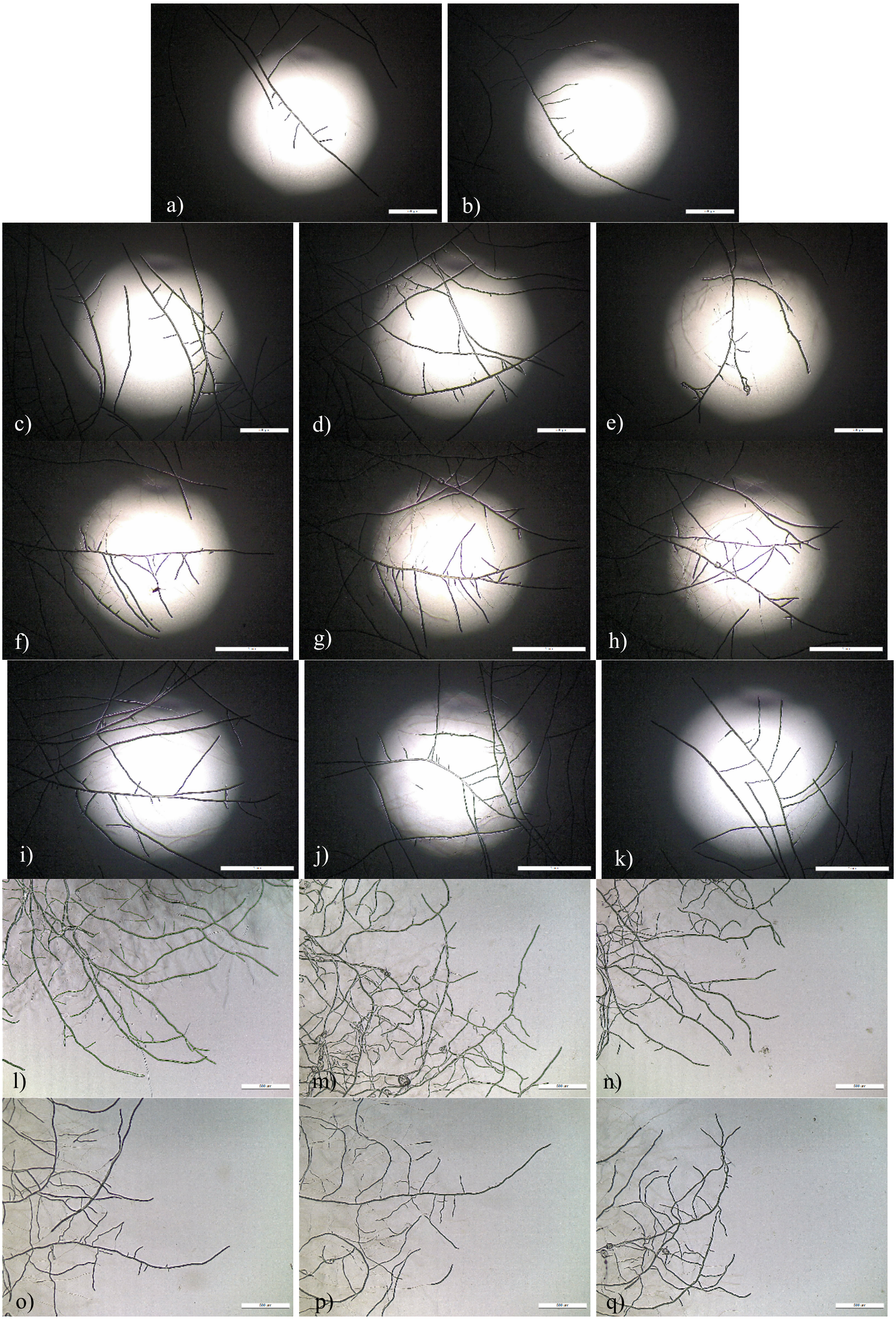

Figure S20. The aspect of hyphal branching of *Colletotrichum acutatum* in a) control water agar, b) acetone, c) GR24 C1, d) GR24 C2, e) GR24 C3, f) SL20 C1, g) SL20 C2, h) SL20 C3, i) SL21 C1, j) SL21 C2, k) SL21 C3, l) SL26 C1, m) SL26 C2, n) SL26 C3, o) SL27 C1, p) SL27 C2, q) SL27 C3 recorded on 2000 μm beginning from the end of the youngest hyphal edge.
